# Nil per os in the management of oropharyngeal dysphagia—exploring the unintended consequences

**DOI:** 10.3389/fresc.2024.1410023

**Published:** 2024-06-18

**Authors:** Michelle Cimoli, Jennifer Gibney, Mathew Lim, Jo Castles, Pedro Dammert

**Affiliations:** ^1^ Speech Pathology Department, Allied Health Division, Austin Health, Heidelberg, VIC, Australia; ^2^ Speech Pathology Department, Nepean Hospital, Penrith, NSW, Australia; ^3^ Dental Services, Alfred Health, Prahran, VIC, Australia; ^4^ Melbourne Dental School, University of Melbourne, Carlton, VIC, Australia; ^5^ Pulmonary and Critical Care Department, Scripps Mercy Hospital Chula Vista, Chula Vista, CA, United States

**Keywords:** dysphagia, ethics, nil per os, aspiration pneumonia, aspiration

## Abstract

Nil per os (NPO), also referred to as Nil by Mouth (NBM), is a health-related intervention of withholding food and fluids. When implemented in the context of a person with dysphagia, NPO aims to mitigate risks of aspiration. However, evidence demonstrating that NPO is beneficial as an intervention for people with dysphagia is lacking. This paper explores the theoretical and empirical evidence relating to the potential benefits and adverse effects of NPO and asserts that NPO is not a benign intervention. This paper argues for applying an ethics framework when making decisions relating to the use of NPO as an intervention for dysphagia, in particular addressing informed consent and a person's right to self-determination.

## Introduction

Oropharyngeal dysphagia is an important health condition to be identified and managed because of the potential consequences on a person's physical health, social participation and well-being (1–3). Problems that affect bolus management and/or airway protection can occur as a result of oropharyngeal dysphagia (4). These difficulties in swallowing may compromise a person's nutrition and hydration (5, 6) as well as impact a person's quality of life (7, 8). Serious consequences such as asphyxiation (5, 9) and aspiration (10) may also result. Oropharyngeal dysphagia is recognized as a risk factor for aspiration pneumonia (5, 11–14).

A variety of intervention approaches are applied in the management and treatment of oropharyngeal dysphagia. These include behavioral, pharmacological, and exercise-based interventional methods and techniques (15). Diet and fluid texture modifications are usually adopted as strategies to compensate for structural and/or biomechanical deficits and facilitate swallowing food and drink (16). The natural textures and consistencies for food and drink may be modified to replace mechanical processes to breakdown the particle size of food or change the viscosity to compensate for changes to the sensorimotor response of swallowing (16). The amount of oral intake may also be limited or completely restricted if a person's swallowing is determined to be unsafe (17).

NPO is an intervention of withholding oral food and/or fluids. Adopted in a range of siutations, NPO may be used as an intervention for people in acute phase of vomiting (18), and is a common intervention for mitigating the risks of aspiration associated with anaesthesia (19). Sedative medications used in anaesthesia depress reflexes and airway protection responses such as the gag and cough reflexes which protect the lungs from stomach contents (19). Hence, fasting (no eating or drinking) prior to surgery or other clinical procedures aims to reduce the volume and acidity of stomach contents to reduce the likelihood of aspiration of stomach acid (via reflux or vomiting) (19).

To prevent complications associated with oropharyngeal dysphagia NPO is implemented to avoid aspiration (5, 17, 20). Despite the common use of NPO as an intervention for oropharyngeal dysphagia, the potential unintended consequences associated with NPO often appear to be overlooked. In this paper, we explore the theoretical and empirical evidence for using NPO as an intervention for oropharyngeal dysphagia. The impact on oral health and the oral biome, nutrition and hydration status, musculoskeletal and respiratory systems, and quality of life associated with NPO are discussed. The ethics of decisions for NPO including key considerations for communicating risk and informed consent are outlined.

### Empirical evidence for NPO

Although the exact frequency of use of NPO to prevent complications associated with oropharyngeal dysphagia such as aspiration is unknown, the inclusion of NPO in guidelines and practice documents relating to the management of oropharyngeal dysphagia (15) suggests NPO is a widespread and accepted approach across a range of clinical settings. The indicators for implementing NPO also appear to be quite varied. A survey study of speech-language pathologists found that the predominant reason for NPO was usually based on judgements that oral intake is unsafe (17) secondary to concerns about aspiration and risk of aspiration (17). Speech-language pathologists made these decisions about a person's swallowing safety on the basis of a clinical swallowing examination, and then advised the patient and the team to proceed with NPO. An evaluation of swallowing function via imaging such as VFSS or FEES is omitted on these occasions. Evaluating swallowing function without using imaging means speech-language pathologists often make decisions for NPO based on additional factors such as level of consciousness, absent swallow, coughing on multiple consistencies, dementia, and a recent or current diagnosis of pneumonia (16).

To the authors' knowledge, there are no randomized controlled trials that directly compare NPO vs. oral feeding for the management of aspiration. Outcomes such as whether NPO prevents the occurrence of aspiration pneumonia have been studied either indirectly, or by observational research designs. A study undertaken to examine the impact of dysphagia screening on rates of aspiration pneumonia in an acute care oncology hospital found NPO did not prevent the occurrence of aspiration pneumonia (21). Similarly, a retrospective cohort study of people with aspiration or penetration on a videofluoroscopic swallowing study found that neither NPO or diet modifications significantly altered the time to first pulmonary event (pneumonia, pneumonitis, or other life-threatening pulmonary illness) when compared to people who were taking food and/or drink orally (22). The researchers found people in the NPO group also developed pneumonia. In a prospective cohort study of stroke survivors conducted by Langdon et al., factors such as transient aspiration of reflux and bacteria secondary to tube feeding were found to be associated with the development of aspiration pneumonia regardless of NPO (23).

### Impact on nutrition and hydration

Withholding food and drink affects a person's nutrition and hydration status. Dehydration due to a reduction in fluid intake by mouth can develop quickly particularly for older people and people whose muscle mass is already reduced (24, 25). Dehydration is especially concerning for people with dysphagia (26) and is associated with higher mortality rates and morbidity in elderly people (27). Moderate hypohydration has been found to affect the heart and can lead to changes in the cardiac system with resultant increases in blood viscosity and haemocrit, hypovolemia and hypotension which place individuals at risk of intravenous and arterial thomobosis, heart arrhythmias and cardiac events, increased risk of falls, altered level of alertness and delirium (28, 29).

Similarly for nutritional intake, in the interim of providing alternative means of delivering nutrition and hydration (e.g., via enteral feeding, intravenously) or resuming oral intake, even relatively short periods of NPO can have a significant impact on nutrition and hydration. Malnutrition is associated with poor clinical outcomes, higher mortality rates and increased length of stay in hospital settings (30, 31). The nutritional and hydrational outcomes of NPO have been explored in conditions such as stroke and critical illness, demonstrating higher mortality in patients with no early enteral nutrition (32–34). NPO has often been shown to be a contributor to hospital-acquired malnutrition (34). A study by Caccialanza and colleagues which examined the association between malnutrition risks and length of stay in hospital identified independent and significant associations between NPO of three or more consecutive days and prolonged hospital stay (35).

### Impact on the musculoskeletal system

Extended periods of fasting and inadequate nutrition can lead to muscle catabolism, where the body starts breaking down muscle tissue for energy resulting in muscle weakness, physical deconditioning, and increased rates of morbidity and mortality (36). These physiological impacts can adversely affect cardiorespiratory function, gastrointestinal function, immunity and wound healing (37). Skeletal muscles are particularly vulnerable to the impacts of malnutrition and dehydration (38) including the muscles involved in oropharyngeal swallowing (38). Problems such as muscle atrophy and changes to the structure and composition of muscles can develop during periods of muscle disuse (38–43). For people who are NPO, the effects of swallowing inactivity and disuse associated with reduced swallowing frequency could feasibly lead to reduced strength in key swallowing musculature including the pharyngeal constrictors and suprahyoid muscles (44). For individuals already malnourished such as older adults, NPO can increase the chances of nutritional sarcopenia and cachexia which in turn can lead to a reduction in the use of the swallowing muscles resulting in dysphagia (37, 44, 45). A study evaluating a treatment protocol for aspiration pneumonia showed that people who were NPO were at high risk of decline in swallowing function (39). NPO in people undergoing treatment for head and neck cancer has been found to be detrimental to long-term functional recovery and swallowing outcomes (46, 47). Research examining the swallowing function and nutritional outcomes of people treated for head and neck cancer has shown those who have periods of NPO and are 100% dependent on enteral feeding for nutrition are less likely to return to full oral intake within a year when compared to those who continue to take some food and drink orally during the course of their treatment (46, 48, 49).

### Impact on the respiratory system

The respiratory system is vulnerable to the adverse effects of dehydration associated with NPO. Humidification is necessary for optimizing the integrity and function of the mucosa lining the upper and lower airway (50, 51). When the mucosa becomes dry, this increases the potential risk for injury, inflammation and infection (52, 53).

Changes in the environment and physiology can affect mucus production as well as the composition and viscosity of mucus (50, 51, 54). When mucus becomes thicker, this can interfere with the normal mechanisms for clearing mucus from the respiratory tract thus leading to the accumulation of mucus (50, 51, 55). When respiratory muscles lose hydration, mucociliary clearance and cough function can become impaired (33, 55, 56). Ciliary function can also be affected when hydration is reduced. Dehydration can cause cilia to move more slowly which affects their capacity to clear mucus and foreign particles from the airway, a phenomenon documented in specific conditions such as cystic fibrosis (50, 51, 55). The accumulation of mucus and impaired ciliary function creates an environment that is more conducive to the colonization of bacteria and viruses thus increasing the risk of respiratory infections (50, 51, 55).

Oral dryness and xerostomia are common symptoms and experiences reported by people who are NPO (57). Oral dryness can interfere with the movement of the oral articulators thus impacting an individual's speech and communication (58). Reduced humidification and hydration of the oropharynx and upper respiratory tract can also affect laryngeal function including voice production (59). Effective vocal function requires both superficial and systemic hydration (60, 61). Changes to hydration and humidification may adversely affect voice production impacting an individual's ability to communicate effectively (59, 60). Hydration in the upper airway is important for optimizing sensory functions of the laryngeal mucosa (50, 62–64).

### Impact on oral health

The impact of NPO on a person's oral health is often overlooked. The oral cavity is the gateway between the external environment and the human body. Therefore, maintenance of oral homeostasis is critical to protect the mouth and prevent disease (65, 66). The impacts of NPO are not isolated to the direct effects on oral health. The link between oral microbial burden and hospital-acquired or aspiration pneumonia is now well-recognized, particularly among those with dysphagia (5, 67–71). NPO can change the microbiota of the mouth triggering dysbiosis and chronic low-grade systemic inflammation leading to the progression of oral and systemic disease (65, 66, 72), and may also be a trigger for cancer and autoimmune disease development (72). The risks to oral health from microbial colonization of the mouth associated with NPO are proportional to the length of time that and individual has reduced oral intake but can occur in relatively short periods (57, 73–75).

NPO interferes with normal oral functions such as chewing and salivation. Chewing helps interrupt the maturation of biofilm (74). Saliva has inherent protective factors related to immunity and has a role in buffering pH in the oral cavity (74). Saliva is essential for the cleaning of oral debris (65, 76). Saliva comprises antimicrobial properties that provide a physiological pH buffer that protects the internal body (65, 76). Saliva also has a major influence on the oral microbiota and assists in maintaining oral homeostasis (65, 76). Reduction in oral functions such as chewing and salivation allow for not only the undisturbed accumulation of plaque but also the maturation of this biofilm and colonization with pathogenic bacterial species (74). In addition, dysbiosis can predispose to other oral infections, such as oral candidosis (77). Reduced salivation may cause, or further exacerbate, pre-existing dry mouth and xerostomia leading to dry mucous membranes and oesophageal mucosa (78–80). For people who are dehydrated, saliva and upper airway secretions may become thick and tenacious, and place an individual at risk of asphyxiation (81).

### Impact on quality of life

Research describes the significant impact of NPO on comfort and quality of life (82). Studies examining the lived experience of NPO have shown that most people who are NPO experience persistent thirst and dry mouth (57). Ho et al. found 58% of people who were NPO in hospital reported thirst even when hydration is maintained via other methods (e.g., intravenous fluids) (83).

People in acute hospitals who are NPO often experience psychological distress. Eating and drinking are social activities and enable us to connect with people in both sickness and health. The rituals of religions, families and communities often revolve around food and drink (84, 85). Several authors have emphasized that aside from providing nutrition and hydration, food and drink provides structure to a person's day (57) and sensory pleasure (85, 86). Eating and drinking also plays an important role in social interactions and celebrations (85, 87, 88). In a study conducted by Carey et al., people who were NPO reported feelings of depression and hopelessness. Participants also felt a loss of control because they were restricted from eating and drinking which impacted their emotional health (57).

The study by Carey et al. also described the tension that can be created in the patient-clinician relationship when restrictive practices such as NPO are implemented (57). Their study revealed that poor communication between healthcare workers and colleagues resulted in people being unnecessarily NPO for extended periods (57). As a result, people who were kept NPO unnecessarily reported a loss of confidence in staff and hospital processes (57). Fear of eating and drinking, and fear of their own saliva was also described by people who were NPO (57). People who were NPO were shown to develop an unrealistic understanding of the risks associated with eating and drinking which impeded their confidence to commence oral intake (57).

Any restrictions (e.g., NPO) and/or modification of oral intake (e.g., texture-modified diets, thickened fluids) in the healthcare setting are considered medical interventions (89–91). Yet, access to food and drink is a basic human right (92) and part of basic care. Arguably, NPO as an intervention for dysphagia contributes to the medicalization of eating and drinking; a non-medical problem such as eating and drinking has come to be conceptualised and treated as a medical problem. As a consequence, a person's right to be the key decision maker in their own health care is transferred from them to the medical profession (93, 94). Being able to decide what is best for oneself is closely linked to motivation, self-esteem and well-being (95).

### Making decisions to proceed with NPO as an intervention for dysphagia

Informed consent is a key element in the ethical and legal provision of healthcare (96). In the situation where a person with dysphagia is presented with NPO as an option for intervention, they must be able to understand the information about NPO (89). Hence, the clinicians involved in the person's care have a duty to support the person's understanding of the situation so the person can make a decision (96). To understand the situation, the person needs to be provided with information about the benefits and harms, alternative interventions, and what is likely to happen if the intervention does not go ahead (89). The person needs to understand that introducing NPO can transfer risk from one physiologic system (e.g., the lungs) to other systems of the body (e.g., musculoskeletal, cardiac, oral microbiome) as these other systems respond to the physiological changes. The harms associated with NPO that extend beyond the physical impacts on the body also need to be presented to the person who is contemplating NPO as an intervention. Arguably, the decision-making and consent process for NPO needs to shift from a focus on disease and medical risks alone to consider the adverse effects on the broader construct of health that includes physical, mental and social well-being (16, 84, 89, 97).

Informed consent requires the person with dysphagia to be an active participant in the decision about interventions (89). There needs to be respect for the person with dysphagia to be an authority in making decisions about their own healthcare (89). The person with dysphagia needs to be encouraged to consider the decision in the context of their preferences and values (89). Studies have shown that people with dysphagia take calculated risks, weighing up the consequences of their symptoms against their values and views, and their desire to remain in control of their lives (86, 97). Extra care is also needed to protect the rights and well-being of vulnerable populations such as children, older persons or people with disabilities who require additional support to participate in decisions about their healthcare.

The decision to proceed with NPO should represent a genuine collaboration between the person with dysphagia and the clinician. The clinician needs to listen closely to the perspectives of the person with dysphagia to consider their social and environmental context in decision-making (86, 88). Clinicians need to bring a sense of humility to these discussions. Humility represents a synchrony between the inward, intellectual perspective of the clinical who acknowledges uncertainty and the limits of the evidence with the outward, social perspective whereby the clinician understands and values the experience of the person receiving healthcare (98). Humility deserves to be a more prominent virtue for all clinicians working with people with dysphagia to aspire to have given that humility is linked to excellence in clinical practice and the effectiveness of therapeutic relationships (98). Humility also allows for the provision of food and drink to be conceptualized within the construct of basic care and an awareness of the unintended consequences of medicalizing eating and drinking (89).

Clinicians are expected to work within an evidence-based practice framework. In the context of decisions about intervention such as NPO, this means being familiar with the evidence revealing the multiple factors that increase the risk of an adverse event associated with aspiration (5, 71). Evidently, there needs to be an active shift away from prioritized aspiration risk as the basis for decisions for NPO (17) given that NPO is not an effective intervention to prevent oropharyngeal secretions colonized by pathogenic bacteria being inhaled into the lungs (80).

Healthcare professionals involved in the care of a person with dysphagia have an ethical duty to advocate for the person's best interests. This includes ensuring that decisions to proceed with NPO are implemented appropriately including monitoring the person's condition to minimize harm. In situations where a person does decide to proceed with NPO as an intervention of dysphagia, ancillary strategies such as care bundles are likely to be important for minimizing the potential deleterious effects of NPO and optimizing outcomes. However, greater scrutiny of widespread practices and policies that describe NPO a default intervention such as dysphagia screening should also be undertaken given the high rate of false positive screening results leading to people being NPO, unnecessarily. The rationale for NPO needs to be documented and communicated (57, 99). Contingencies need to be in place to avoid people being unintentionally kept NPO due to communication breakdowns between healthcare workers (57, 99). To that end, the number of days a person is NPO represents a valuable outcome measure that has the potential to inform the clinical reasoning process. When interrogated as part of a larger data set, the number of days NPO may also tell a story about patterns of clinical reasoning within a specific patient cohort or within a group of clinicians.

## Conclusion

The dearth of strong evidence to support the use of NPO as a default intervention warrants a prudent, if not a cautious approach when considering NPO for a person with dysphagia. In pursuit of excellence in clinical practice, the virtue of humility should be more prominent when making decisions about using NPO as an intervention for dysphagia-related consequences such as aspiration. In the situation where a person decides to proceed with NPO as an intervention for oropharyngeal dysphagia, ancillary support should be adopted to limit the deleterious impacts of this high-risk intervention. More research is needed to examine the effects and the experience of NPO. In the interim, the use of NPO even for a short time requires a more intentional approach to mitigating the potential harms of this intervention.
